# Estimating Burden of Disease Among Blind Individuals With Non-24-Hour Sleep-Wake Disorder

**DOI:** 10.3389/fneur.2020.605240

**Published:** 2021-01-21

**Authors:** Lauren Van Draanen, Changfu Xiao, Mihael H. Polymeropoulos

**Affiliations:** Vanda Pharmaceuticals Inc., Washington, DC, United States

**Keywords:** non-24 hour sleep-wake disorder, blind & visually impaired people, HRQOL–health-related quality of life, burden of disease (BOD), quality of life, non-24

## Abstract

**Purpose:** To quantify the burden of disease in blind patients with Non-24-H Sleep- Wake Disorder (N24HSWD), utilizing longitudinal sleep diary data. N24HSWD is a circadian disorder characterized by a cyclical pattern of aberrant circadian and sleep-wake cycles that are associated with increased frequency of sleep episodes during the school/work day hours. Daytime sleep episodes would be predicted to decrease the opportunity for school/work participation, significantly impacting the quality of life of the patient.

**Methods:** We used the sleep diary data of daytime sleep from a period of ~90 days in blind individuals that presented with a sleep complaint. These subjects were identified from a group of blind individuals with N24HSWD (*n* = 121) and a control group of blind individuals without N24HSWD (*n* = 57).

**Results:** N24HSWD patients had more frequent and longer episodes of daytime sleep as compared to a control group. Using duration of daytime sleep as a surrogate for defining a healthy or unhealthy day, N24HSWD patients also had significantly fewer healthy days, defined by daytime sleep free days (DSFD), days without a sleep episode between 9:00 a.m. and 5:00 p.m, as compared to the control group.

**Conclusion:** Daytime sleep free day (DSFD) is a useful and specific measure of disease burden in patients with N24HSWD and it is predicted to be correlated with the standardized HRQOL-4, Healthy Days measurement.

## Introduction

Non-24-Hour Sleep-Wake Disorder (N24HSWD) is a rare circadian disorder that affects both sighted and blind patients but is highly prevalent among individuals who are totally blind and lack light perception (1). N24HSWD results from a misalignment of the body's internal circadian timing system with the external 24-h clock. In the human brain, a complex circadian timing system residing in the suprachiasmatic nucleus (SCN) sets in motion the coordinated expression of a myriad of physiological processes, including the sleep-wake cycle, hormonal secretion, feeding behavior, and metabolic processes, among many. The endogenous timing system has a beat cycle of a little longer than 24 h and in blind individuals, it is around 24.20 h (2). This endogenous circadian system is reset every day through the perception of light through the retina, therefore maintaining a periodicity of 24 h (1). In some totally blind individuals with no light perception, this ability to reset the internal circadian system may be compromised (1, 3). Without the ability to reset the internal circadian system, the disorder may manifest itself as a constant and progressive misalignment between the internal and external clock. The result is a sleep wake cycle that is misaligned with social norms and in turn, results in a N24HSWD patient sleeping and waking at the wrong times of the day for extended periods, synchronizing for a brief period and then the cycle repeats in a perpetual manner. Patients with N24HSWD suffer chronically from the condition, which has significant impact on social and occupational functioning (4). For the extended periods when the endogenous cycle creates daytime sleep propensity, patients cannot resist falling asleep in the middle of the day, a situation often incompatible with successful performance in school and work activities (5). While differences may exist regarding the prevalence of naps between cultures and age groups, consistent, extended periods of sleep during daytime hours would pose difficulties to function within a normal society. Businesses, schools, stores, public transportation and other institutions are likely to provide their services during primarily daytime hours. With the cyclical nature of N24HSWD, there will be a number of days during where the circadian cycle is at its extreme inverse of the normal 24-h day, with the propensity for sleep occurring during the day. For individuals with N24HSWD, this sleep propensity is equivalent to a “normal” individual's night, not simply a daytime nap or rest. In contrast, naps in the general population occur as sleep propensity during the day without the absence of sleep propensity during the night. In this study we aim to quantify the burden of disease in N24HSWD patients, by utilizing longitudinal sleep diary data and transforming them into a surrogate measurement of healthy days in a given 30-day period. We refer to this measurement as daytime sleep free days (DSFD), measured through different thresholds of sleep between 9:00 a.m. and 5:00 p.m. DSFD is expressed as daytime sleep free days in a 30-day period. This transformation allows us to compare this measure with unhealthy days as measured by the Centers for Disease Control (CDC) Behavioral Risk Factor Surveillance System Health Related Quality of Life (HRQOL-4) questionnaire in the general population (6). In summary, this paper compares DSFD between blind patients with and without N24HSWD from a controlled study. In addition, it compares the unhealthy days transformed using the DSFD measure in the controlled study population of blind patients with and without N24HSWD, with the number of unhealthy days from U.S. CDC HRQOL survey data in the general U.S. population. While further studies will be needed to validate the DSFD as a measure of disease burden in N24HSWD, our work establishes DSFD as a specific tool to allow for the study of the impact of N24HSWD in patients with this severe and debilitating chronic condition.

## Materials and Methods

### Cohort Description

The patients in this study are a sequential cohort of blind patients with a sleep complaint that participated in a clinical program, aimed at the development of a treatment for N24HSWD. The study, known as the Safety and Efficacy of Tasimelteon (SET) study was conducted between 2010 and 2013 in sites in the United States, France and Germany (1). Participating individuals agreed to the procedures of the study by signing an informed consent, which was approved by the appropriate Institutional Review Boards (IRB). In total for this study, we are including 178 blind patients with a sleep complaint, of whom 121 were shown to have N24HSWD and 57 did not and are used as controls. Demographic data for the participants is shown in Table 1.

**Table 1:** Characteristics of the control and Non-24-Hour Sleep-Wake Disorder subjects from the SET (1) study.

	**Disease status**	
**Characteristic**	**Control**	**N24HSWD**
	**(*N* = 57)**	**(*N* = 121)**
Sex		
Male	20 (35%)	72 (60%)
Female	37 (65%)	49 (40%)
Fisher *p*-value: 0.0036		
Age (years)		
Mean (SD)	46.9 (13.45)	50.8 (13.26)
*T*-test *p*-value: 0.2960		
Race		
American Indian or Alaska Native	2 (3.5%)	1 (1%)
Asian	1 (1.8%)	1 (1%)
Black or African American	9 (16%)	18 (15%)
Native Hawaiian or other Pacific Islander	1 (1.8%)	0 (0%)
Other	0 (0%)	3 (2%)
White	44 (77%)	98 (81%)
Fisher *p*-value: 0.2993		
Weight (kg)		
Mean (SD)	46.86 (13.45)	50.82 (13.27)
T-test p-value: 0.2960		
BMI (kg/m^2^)		
Mean (SD)	27.8 (3.79)	28.15 (3.9)
*T*-test *p*-value: 0.5581		
Tau (hours)		
Mean (SD)	23.9 (0.04)	24.54 (0.27)
*T*-test *p*-value: <0.0001		
Average length of sleep diary collection (days)		
Mean (SD)	64.01 (39.2)	92.82 (49.71)
*T*-test *p*-value: <0.0001		

The SET/RESET trials were completed in 27 US and six German clinical research centers and sleep centers. The studies were approved by central and local Institutional Review Boards (the Ethics Committees that reviewed SET protocols in Germany were Ethikkommission der Ärztekammer Hamburg and Landesamt für Gesundheit und Soziales Berlin; the Ethics Committees that reviewed the SET or RESET protocols, or both, in the USA were Chesapeake Research Review, the Institutional Review Board New York Eye and Ear Infirmary, Minneapolis Medical Research Foundation Human Subjects Research Committee, Partners Human Research Committee, the Research Compliance Office Stanford University, and St Luke's Hospital Institutional Review Board). These studies were done in accordance with Good Clinical Practice, as required by the US Food and Drug Administration and German regulatory bodies (where applicable), and in agreement with the Declaration of Helsinki.

### Sleep Diaries/CDC HRQOL-4 Questionnaire

For the blind cohort from the SET/RESET study, daily sleep diary data were collected through an interactive voice response system (IVRS) (1). Average length of sleep diary collection for the two groups is shown in the demographics table (Table 1). The blind population from the SET/RESET study did not complete the HRQOL-4 questionnaire; instead, data transformations (as described below) were used to define healthy days for this population.

The CDC population completed the HRQOL-4 Questionnaire. The HRQOL-4 Questionnaire consists of four standardized questions developed by the CDC in 1993 (6, 7). The four questions are used to evaluate overall health, physical and mental health. After the first overall health question, the following three questions are used to evaluate how physical health, mental health and both physical and mental health were “not good” in the last 30 days (Table 2). These questions have been validated across the United States and various patient populations as a robust method to measure a person's quality of life and determine how many days in a month they feel that their physical health, mental health or both affected their days (8–10). In the CDC data from 2013, 2014 and 2015, 1,397,893 individuals participated in the survey. The CDC data is randomly collected from individuals across the United States through phone call interviews where participants are asked the HRQOL questionnaire, in addition to other CDC questions.

**Table 2:** CDC Behavioral Risk Factor Surveillance System Questionnaire (HRQOL) (3).

**Questions:**	**Answers:**	**Score:**
**Section 1: health status**		
1.1: Would you say that in general your health is:	Read:	
	Excellent	1
	Very good	2
	Good	3
	Fair	4
	Poor	5
	Not Read:	
	Don't know/Not sure	7
	Refused	9
**Section 2: Healthy Days- Health-Related Quality of Life (HRQOL)**		
2.1: Now thinking about your physical health, which includes physical illness and injury, for how many days during the past 30 days was your physical health not good?		
	Number of Days	
	None	8 8
	Don't know/Not sure	7 7
	Refused	9 9
2.2: Now thinking about your mental health, which includes stress, depression, and problems with emotions, for how many days during the past 30 days was your mental health not good?		
	Number of Days	
	None	8 8
	Don't know/Not sure	7 7
	Refused	9 9
2.3: During the past 30 days, for about how many days did poor physical or mental health keep you from doing your usual activities, such as self-care, work, or recreation?		
	Number of Days	
	None	8 8
	Don't know/Not sure	7 7
	Refused	9 9

The HRQOL instrument is used to measure the subjective quality of life for people across diverse geographies (11–19). The CDC standardized a HRQOL questionnaire in the early 1990s and it has been validated across the United States and the globe (12, 14). The basic questionnaire includes four questions related to mental and physical health, and it has been expanded across various disease states and physical and mental conditions. Adaptations have been created in many languages, by large organizations such as the World Health Organization and various countries and states across the world.

### Data Transformations

To assess the disease burden of N24HSWD on the daily social/working life, the daily sleep diary data reported from 9:00 a.m. to 5:00 p.m. in an interactive voice recording system (IVRS) were studied. We defined “daytime” as the time between 9:00 a.m. and 5:00 p.m., as these are typical hours used for most work or school days. Therefore, extended sleep duration during that time would likely interfere with standard work or school activities. (4) While any length of a sleep episode is disruptive, for the purposes of the work presented in this manuscript we developed four categorical thresholds that defined Daytime Sleep Free Days (DSFD) as days with no daytime sleep, less than a half an hour of sleep, <1 h of sleep or <2 h of sleep. Given the cyclical nature of the disorder, patients are expected to cycle in and out of phase over time, and as such, experience days and nights of sleep disruption followed by brief periods of circadian phase alignment and sleep wake normalcy. In order to increase the sensitivity of detection of a DSFD difference between N24HSWD patients and controls, we focused our analysis on the worst quartile of the sleep diary collection for both patient and control groups. This measure reflects the periods in which the sleep wake cycle is mostly out of phase, and is referred to as Upper Quartile Daytime Sleep Duration (UQ-dTSD) (Table 3). UQ-dTSD data was assessed to determine the probability of DSFD per day under different thresholds. The probability of DSFD per day was transformed to the actual number of DSFD within 30 days (Table 4). DSFDs were calculated using four different threshold criteria. A day without any sleep, a day with less than half an hour of sleep, a day with <1 h of sleep, or a day with <2 h of sleep (Table 3). These thresholds were used to determine the number of DSFDs out of a 30-day month. The remaining (non-DSFD) number of days within the 30-day period were compared to the unhealthy days per 30 days from HRQOL-4 (Table 5). The length of in-phase or out-phase, determined by each patient's circadian shifting speed (Tau), can range from a couple of weeks to a couple of months. The diary collection period in Table 1 is somewhat limited, and may only cover approximately one or 1.5 cycles (i.e., two in-phases and one out-phase). So the DSFD, transformed from UQ-dTSD, more reasonably represents the daytime social/work impairment of N24HSWD patients when they are experiencing the out-phase period of their endogenous circadian rhythm.

**Table 3:** Summary of the daytime sleep duration (9:00 a.m.−5:00 p.m.) by disease status.

	**Disease status**		
**Daytime sleep duration (dTSD)**	**Control**	**N24HSWD**	***p-*Value**^**c**^
	**(*N* = 57)**	**(N=121)**	
dTSD^a^ (hours) (SD)	0.38 (0.45)	0.60 (0.57)	0.0006
UQ_dTSD^b^ (hours) (SD)	1.04 (0.95)	1.85 (1.49)	<0.0001

a *dTSD: average daytime sleep duration (hours) between 9:00 a.m. and 5:00 p.m*.

b *UQ_dTSD: upper quartile daytime sleep duration (hours)*.

c *p-values are based on an ANCOVA model*.

**Table 4:** Summary of the daytime sleep free days (DSFD)^a^.

	**Disease status**		
**Threshold for daytime sleep**	**Control**	**N24HSWD**	***p-*Value**^**c**^
**free days (DSFD)**	**(*N* = 57)**	**(*N* = 121)**	
0 hours of daytime sleep			
DSFD/Total^b^: mean (SD)	0.31 (0.37)	0.18 (0.28)	0.0050
DSFD/month (days)	9	5	
0.5 hour of daytime sleep			
DSFD/Total: mean (SD)	0.42 (0.37)	0.24 (0.31)	0.0006
DSFD/month (days)	13	7	
1 hour of daytime sleep			
DSFD/Total: mean (SD)	0.58 (0.38)	0.37 (0.33)	<0.0001
DSFD/month (days)	17	11	
2 hours of daytime sleep			
DSFD/Total: mean (SD)	0.78 (0.35)	0.60 (0.36)	<0.0001
DSFD/month (days)	23	18	

a *DSFD are the number of daytime sleep free days in a 30-day period. Four different thresholds define daytime sleep free days: 0, 0.5, 1, and 2 h of daytime sleep between 9:00 a.m. and 5:00 p.m*.

b *DSFD/Total is the number of daytime sleep free days that are at or below the threshold divided by the total number of diary recorded days*.

c *p-values are based on an ANCOVA model*.

**Table 5:** Comparison of daytime sleep free days in the SET study and CDC Health Related Quality of Life (HRQOL-4) survey responses in the U.S. population (blind and non-blind).

	**CDC survey data**^**a**^		**SET screening data (1 h DSFD threshold)**		
	**Non-blind**	**Blind**	**Control**	**N24HSWD**	***p-*Value**^**c**^
	**(*N* = 1,266,031)**	**(*N* = 67,937)**	**(*N* = 57)**	**(*N* = 121)**	
**HRQOL-4 unhealthy days**					
Physical–unhealthy days (SD)	3.9 (8.40)	11.4 (12.56)			
Mental–unhealthy days (SD)	3.1 (7.29)	8.0 (11.38)			
Physical and/or mental unhealthy days (SD)	4.8 (8.94)	11.2 (12.27)			
Non-DSFD (SD)^b^			12.7 (11.46)	19.0 (10.15)	^*^see Table 3
**Statistical Comparison**^d^					
1. Blind vs. N24HSWD		✓		✓	<0.0001
2. Blind vs. Control		✓	✓		0.1025
3. Non-Blind vs. N24HSWD	✓			✓	<0.0001
4. Non-Blind vs. Control	✓		✓		<0.0001

a *The population is based on U.S. CDC survey data (2013–2015)^c^*.

b *Unhealthy Days for SET subjects were defined as days where study subjects slept more than 1 h between 9:00 a.m. and 5:00 p.m*.

c *p-values are based on the non-parametric Wilcoxon-Mann–Whitney test*.

d *Check mark symbols indicate the populations compared for the p-value in the last column*.

### Statistical Analysis

An ANCOVA model adjusted for demographic variables (sex, age, race, and BMI) was used to assess the difference of daytime sleep duration (dTSD), UQ-dTSD, and DSFD between N24HSWD patients and controls. When comparing to the U.S. CDC HRQOL-4 data, a non-parametric test of Wilcoxon–Mann–Whitney was used. The *p*-values were displayed to assess the significance at 0.05 levels.

All analyses were conducted using SAS 9.4 (SAS Institute Inc., Cary, NC, USA).

## Results

The N24HSWD patients showed a statistically significant higher dTSD and UQ-dTSD within the normal working/school time period as compared to the blind control cohort from the SET/RESET study (Table 3). dTSD and the UQ_dTSD for control patients was 0.38 or 1.04 h, while it was significantly higher at 0.60 or 1.85 h for N24HSWD patients (*p*-values are 0.0006 and <0.0001, respectively). Blind patients with N24HSWD had more sleep burden during the working /school time than the control cohort of blind patents without N24HSWD. N24HSWD patients had consistently lower DSFD than the control cohort of blind patents without N24HSWD for all thresholds (Table 4, all *p* < 0.01).

We considered a day with 1 h or more of daytime sleep during the working/social time as an “unhealthy day” within the SET/RESET study population of blind patients with a sleep complaint. Table 5 summarizes the comparisons between the SET/RESET study populations of blind patients with and without N24HSWD and the U.S. CDC survey populations (2013–2015) regarding the HRQOL-4 survey of “unhealthy days.” Blind patients with N24HSWD had a significant higher number of unhealthy days than the blind population in the CDC data (*p* < 0.0001). In contrast to the N24HSWD cohort, blind patients without N24HSWD (control cohort) showed a similar number of unhealthy days as compared to the blind population in the CDC data (12.7 vs. 11.2 respectively, *p* = 0.1025). Finally, blind patients with N24HSWD and without N24HSWD (control cohort) had much higher numbers of unhealthy days than the non-blind population in CDC (both *p* < 0.0001).

## Discussion

People with self-reported blindness in the U.S. CDC survey population had approximately the same number of unhealthy days as the control patients from the SET study when we applied the definition of >1 h of daytime sleep as an unhealthy day in that population (6). N24HSWD patients had significantly more unhealthy days than both the control population and the blind and non-blind populations of the U.S. CDC survey populations (1, 8). This demonstrates the additional burden that blind patients with N24HSWD are experiencing, specifically due to the sleep-wake cycle misalignment induced by the condition.

N24HSWD results in significant impairment, in part due to daytime sleep propensity that results in a number of days within a given month where daytime sleep occurs during the 9:00 a.m. to 5:00 p.m. timeframe (4). Our study shows that the daytime sleep propensity and resulting functional impairment is more significant among N24HSWD patients than in patients of the control cohort, despite the fact that they share similar baseline characteristics and present with a similar sleep complaint.

Under the assumption that 1 h of daytime sleep significantly affects function, we defined DSFD as days with <1 h of daytime sleep. Under this condition N24HSWD patients experienced 11 DSFD, compared to patients without N24HSWD who presented with 17 DSFD (*p* < 0.0001). The number of non-DSFD became the unhealthy days, indicating 1 h or more of daytime sleep. This exhibits the additional difficulties that patients with N24HSWD face. The N24HSWD patients had significantly more unhealthy days than the control cohort did (19 vs. 13, respectively, *p* < 0.0001).

In order to examine how the magnitude of DSFD compared with impairment in the population, we used the annual U.S. CDC data collected through the Health Related Quality of Life survey (HRQOL) (6, 7). The HRQOL survey, asks individuals a set of standard questions in order to determine, subjectively, how many days in the last 30 days the person would define as feeling “healthy” or “unhealthy.” This questionnaire has been validated across many different disease states and populations as a robust standard for evaluating overall health of an individual or population (12–19). For the purpose of this analysis, we made the assumption that Healthy Days as defined in the HRQOL are equivalent to DSFDs. The HRQOL is utilized to measure a person's subjective quality of life. We used CDC data from 2013 to 2015 to analyse “Healthy/Unhealthy Days” in the general population, as well as the self-reported visually impaired subpopulation. N24HSWD patients experienced fewer “healthy days” in a 30-day period than the general U.S. population in the CDC survey (Table 5) (6). This is to be expected, with N24HSWD patients facing the burden of total blindness as well as a non-entrained circadian rhythm (1, 3, 4). Additionally N24HSWD patients experienced fewer “healthy days” in a 30-day period than patients with self-reported visual impairment in the CDC survey. In addition to the significant difference between the DSFD for N24HSWD patients and control cohort patients from the SET study, there is a significant difference between the “healthy days” for N24HSWD patients and the visually impaired U.S. CDC population. This again exhibits the additional burden of disease that N24HSWD presents to patients. The “healthy days” that control patients and CDC visually impaired patients experienced were not statistically significant, as we might expect. Control cohort patients were found to have an entrained circadian rhythm, despite sleep complaints, which may be a reason that they appear more similar to the CDC visually impaired patients, and they are different from N24HSWD patients. We conclude that DSFD can be a useful measure of impairment in patients with N24HSWD disorder and could allow for the evaluation of the impact of treatments aimed at improving the symptoms of this disorder.

There are several limitations of this work that may impact the conclusions. First, N24HSWD patients did not receive the HRQOL questionnaire and therefore direct comparison cannot be made. The N24HSWD patients had similar baseline parameters (Table 1), with the exception of the male/female ratio and the length of sleep diary collection, although we believe that these parameters are unlikely to affect our conclusions. The visual impairment described in the CDC population, likely includes patients who are not totally blind, in contrast to the totally blind population of the SET study. A potential confounder of this analysis is the prevalence of daytime napping in the general population. This has not been well-quantified in the literature, as it varies by culture and age. Given the published differences in daytime sleep propensity caused by N24HSWD, and the cyclical, often uncontrollable nature of the condition, we believe this analysis is still meaningful in evaluating the burden of disease of N24HSWD and the likely impacts of daytime sleep on this population. Notwithstanding these limitations, it is likely that directionally our conclusions are valid, while the magnitude of the comparative observations will be less certain. Future studies could be designed to prospectively quantify the impact of N24SWD on quality of life and compare that with populations with varying degrees of visual impairment as well as the general population.

In conclusion, this paper examines the sleep data from the largest conducted study in blind patients with and without N24HSWD, and compares this blind population to a large CDC survey of individuals who completed the HRQOL questionnaire. Using sleep data from the study in blind patients, we developed a novel methodology to quantify impairment caused by extended daytime sleep episodes on social and occupational functioning. The analysis of the daytime sleep duration in the blind populations was used to create a threshold for “healthy days” and that data was compared to CDC data of healthy days in a general population. Our work found that significantly more blind individuals with N24HSWD had a deficit of healthy days as compared to blind individuals without N24HSWD, and as compared to the general CDC population. This analysis demonstrates the additional burden faced by blind individuals with N24HSWD and the likely impact of N24HSWD on daytime functioning in social and occupational settings.

## Data Availability Statement

The original contributions presented in the study are included in the article/supplementary materials, further inquiries can be directed to the corresponding author/s.

## Ethics Statement

The studies were approved by central and local Institutional Review Boards (the Ethics Committees that reviewed SET protocols in Germany were Ethikkommission der Ärztekammer Hamburg and Landesamt für Gesundheit und Soziales Berlin; the Ethics Committees that reviewed the SET or RESET protocols, or both, in the USA were Chesapeake Research Review, the Institutional Review In review Board New York Eye and Ear Infirmary, Minneapolis Medical Research Foundation Human Subjects Research Committee, Partners Human Research Committee, the Research Compliance Office Stanford University, and St Luke's Hospital Institutional Review Board). The patients/participants provided their written informed consent to participate in this study.

## Author Contributions

All authors listed have made a substantial, direct and intellectual contribution to the work, and approved it for publication.

## Conflict of Interest

LV, CX, and MP are employed by Vanda Pharmaceuticals Inc.
